# Stretch-Induced Activation of Pannexin 1 Channels Can Be Prevented by PKA-Dependent Phosphorylation

**DOI:** 10.3390/ijms21239180

**Published:** 2020-12-02

**Authors:** Ximena López, Rosalba Escamilla, Paola Fernández, Yorley Duarte, Fernando González-Nilo, Nicolás Palacios-Prado, Agustín D. Martinez, Juan C. Sáez

**Affiliations:** 1 Departamento de Fisiología, Facultad de Ciencias Biológicas, Pontificia Universidad Católica de Chile, Santiago 8331150, Chile; nicopalacios@uc.cl; 2 Facultad de Ciencias, Instituto de Neurociencias and Centro Interdisciplinario de Neurociencias de Valparaíso, Universidad de Valparaíso, Valparaíso 2381850, Chile; rescamhdz@yahoo.com (R.E.); paolafernandez.bq@gmail.com (P.F.); yorley.duarte@unab.cl (Y.D.); danilo.gonzaleznilo@gmail.com (F.G.-N.); agustin.martinez@uv.cl (A.D.M.); 3 Center for Bioinformatics and Integrative Biology, Facultad de Ciencias de la Vida, Universidad Andrés Bello, Av. República 330, Santiago 8370146, Chile

**Keywords:** protein phosphorylation, site-directed mutation, dye uptake, adenosine, cAMP

## Abstract

Pannexin 1 channels located in the cell membrane are permeable to ions, metabolites, and signaling molecules. While the activity of these channels is known to be modulated by phosphorylation on T198, T308, and S206, the possible involvement of other putative phosphorylation sites remains unknown. Here, we describe that the activity of Panx1 channels induced by mechanical stretch is reduced by adenosine via a PKA-dependent pathway. The mechanical stretch-induced activity—measured by changes in DAPI uptake—of Panx1 channels expressed in HeLa cell transfectants was inhibited by adenosine or cAMP analogs that permeate the cell membrane. Moreover, inhibition of PKA but not PKC, p38 MAPK, Akt, or PKG prevented the effects of cAMP analogs, suggesting the involvement of Panx1 phosphorylation by PKA. Accordingly, alanine substitution of T302 or S328, two putative PKA phosphorylation sites, prevented the inhibitory effect of cAMP analogs. Moreover, phosphomimetic mutation of either T302 or S328 to aspartate prevented the mechanical stretch-induced activation of Panx1 channels. A molecular dynamics simulation revealed that T302 and S328 are located in the water–lipid interphase near the lateral tunnel of the intracellular region, suggesting that their phosphorylation could promote conformational changes in lateral tunnels. Thus, Panx1 phosphorylation via PKA could be modulated by G protein-coupled receptors associated with the Gs subunit.

## 1. Introduction

Pannexins (Panxs) are glycoproteins expressed in vertebrates, which have sequence homology with the proteins that form gap junction channels and hemichannels in invertebrates, called innexins [1,2,3]. So far, three Panx subtypes have been reported (Panx1, Panx2, and Panx3); Panx1 is expressed ubiquitously [2]. It has recently been shown that channels formed by Panx1 result from the oligomerization of seven monomers [4]. Each monomer has a membrane topology similar to connexin-based channel subunits [5]. Activated Panx1 channels are non-selective and allow the transfer of charged small molecules, such as ATP and DAPI [6,7]. Therefore, regulated gating is necessary to maintain ion balance and cell integrity.

Panx1 channels can be activated in physiological or pathological conditions, describing two states of permeability and conductance depending on the type of stimulation [8,9]. Panx1 participates in purinergic signaling through the release of ATP in different cell types, including neurons, astrocytes, erythrocytes, endothelial cells, gustatory cells, and immune system cells [6,10,11,12]. The functional role of Panx1 channels is cell-type specific, e.g., in macrophages, neurons, and astrocytes. Pathological conditions induce Panx1 channel activity via P2X7 receptor (P2X7R) [13,14] and promote apoptosis through the activation of NLRP1 and NLRP3 inflammasomes [15,16,17].

In different cell types, activation of Panx1 channels has been experimentally induced by a high concentration of extracellular K^+^, ischemia, electrical stimulation, an increase of cytoplasmic Ca^2+^ concentration, proteolysis of their C-terminal domains [11,14,15,18,19,20,21,22,23,24,25,26,27], and mechanical stress induced by shear stress [6,28,29,30,31]. These stimuli can lead to the modulation of Panx1 channel activity by post-translational modifications such as phosphorylation or S-nitrosylation [32,33].

Panx1 channel phosphorylation plays a fundamental role in acute vascular inflammation [33]. In this process, a signaling cascade leading to the recruitment of the Src family of tyrosine kinases (SFKs) is activated and phosphorylation of Panx1 in tyrosine 198 results in increased activity of Panx1 channels. Additionally, Panx1 channels can also be activated through a SFK-dependent pathway during anoxia or NMDA receptor activation in CA1 pyramidal neurons [34]. By using an interfering peptide against the consensus phosphorylation site for SFKs in Panx1, tyrosine 308, anoxia-induced depolarization is prevented. Therefore, the activation of NMDA receptors during anoxia recruits the SFKs, which allow the activation of Panx1 channels through the phosphorylation of tyrosine 308, leading to sustained neuronal depolarizations. However, Panx1 phosphorylation may also induce a decrease in channel activity, e.g., currents associated with exogenous Panx1 channels in HEK-293 cells are attenuated by nitric oxide [35]. This is explained by the activation of guanylate cyclase and subsequent reduction in Panx1 channel activity due to phosphorylation of S206 by cGMP-dependent kinase (PKG).

Despite the above, it is unknown whether the activity of Panx1 can be affected by phosphorylation of other amino acid residues. The present work was undertaken to study the regulation of Panx1 channels by adenosine in a mechanism that involves an increase in cytoplasmic cAMP and subsequent activation of PKA [36]. We found two previously unidentified PKA target sites that modulate the activity of Panx1 channels.

## 2. Results

### 2.1. Dye Uptake Mediated by Pannexin 1 Channels is Inhibited by Adenosine and cAMP Analogs

**Figure 1:**
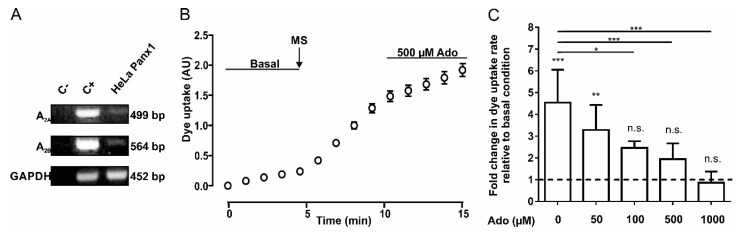
Adenosine reduces the activity of Panx1 channels. (**A**) RT-PCR of A_2A_ and A_2B_ adenosine receptors in HeLa Panx1 cells. The PCR reaction without template DNA was used as a negative control (C-) and the Jurkat cell cDNA was used as a positive control (C+). GAPDH was used as a constitutive expression control and its amplicon of 452 bp was used as loading gel control. (**B**) Time course of DAPI uptake in HeLa Panx1 cells at basal condition, after mechanical stretch (MS, the arrow indicates when it was applied) induced by dropping 6 mL, and during treatment with 500 µM adenosine (Ado). (**C**) Normalized dye uptake rate in HeLa Panx1 cells after mechanical stretch and subsequent treatment with different concentrations of adenosine. Statistical analysis was performed between each experimental group (connecting lines) and comparing each group with baseline (symbols above each bar). Note: * *p* < 0.05, ** *p* < 0.005, *** *p* <0.001; n.s., not significant. Each value corresponds to the average ± standard error of a total of three to seven independent experiments.

We determined whether HeLa Panx1 cells express the mRNA of adenosine receptors A_2A_ and A_2B_. To verify this, we performed RT-PCR to detect the mRNA of both receptors; as a positive control, we used cDNA from Jurkat cells. The products were separated in agarose gels (Figure 1A). The top and central panels show representative PCR products of adenosine receptors A_2A_ (499 bp) and A_2B_ (564 bp), respectively. Results obtained using a negative control (C-, without template DNA), positive control (C+, cDNA from Jurkat cells), and cDNA from HeLa Panx1 cells are shown. Although we observed faint bands in HeLa Panx1 cells, we concluded that mRNAs of A_2A_ and A_2B_ receptors were present.

To study Panx1 channel activity, we performed DAPI uptake assays in HeLa Panx1 cells. Upon mechanical stretch, HeLa-P cells did not show significant changes in DAPI uptake rate, but HeLa Panx1 cells showed a significant increase in the DAPI uptake rate compared with basal conditions (Figure 1B and Supplementary Figure S1B,C). Although the dye uptake of HeLa-P cells was much less sensitive to mechanical stretch, a small and variable baseline uptake of DAPI was detected, which could be explained by very low expression of endogenous connexin45 hemichannels [37]. After performing the mechanical stretch protocol, cells were treated with 500 µM adenosine for 5 min, causing a decrease in the DAPI uptake rate (Figure 1B), which showed a concentration-dependent relationship (Figure 1C).

**Figure 2:**
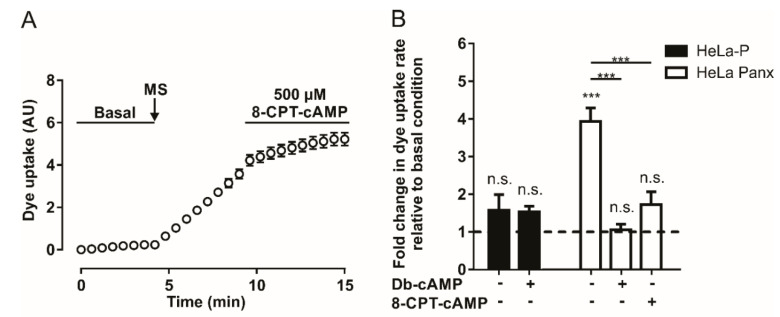
The cAMP analogs reduce mechanical stretch-induced activity of Panx1 channels. (**A**) Temporary course of DAPI uptake in HeLa-Panx1 cells at basal condition, after mechanical stretch (MS, arrow) and during treatment with 500 µM 8-CPT-cAMP. (**B**) Normalized dye uptake rate in HeLa-P (black bars) and HeLa Panx1 (white bars) cells subjected to MS and subsequently treated with Db-cAMP or 8-CPT-cAMP. Statistical analysis in B was performed between each experimental group (connecting lines) and comparing each group with baseline (symbols above each bar). Note: *** *p* < 0.001; n.s., not significant. Each value in B corresponds to the average ± standard error of a total of four to ten independent experiments.

Since A_2A_ and A_2B_ adenosine receptors are G_αs_ protein-coupled receptors, their activation should promote an increase in intracellular cAMP concentration [38]. To evaluate whether an increase in intracellular cAMP affects mechanical stretch-induced activation of Panx1 channels, we used two cell-permeant cAMP analogs (8-CPT-cAMP and Db-cAMP). Application of 500 µM of 8-CPT-cAMP after mechanical-stretch-dependent activation induces a reduction in the rate of DAPI uptake (Figure 2A). Figure 2B shows the average of four to ten experiments with 8-CPT-cAMP or Db-cAMP; both analogs diminished the rate of DAPI uptake after mechanical stretch-induced activation of Panx1 channels. These experiments show that the activity of Panx1 channels is inhibited by a mechanism triggered by adenosine or by an increase in intracellular cAMP.

### 2.2. PKA Is Involved in the Inhibition Induced by Db-cAMP on Mechanical Stretch-Induced Activation of Panx1 Channels

**Figure 3:**
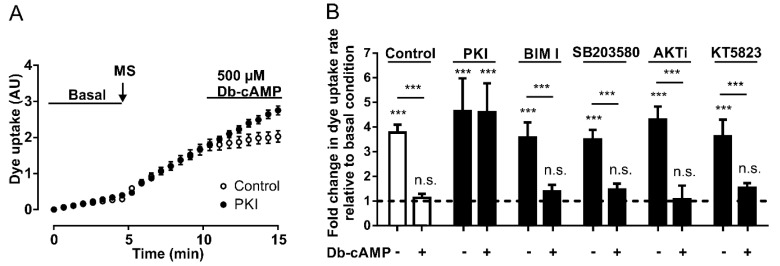
The inhibitory effect of Db-cAMP on Panx1 channel activity depends on PKA. (**A**) Temporary course of DAPI uptake in HeLa-Panx1 cells at basal condition, after mechanical stretch (MS, arrow) and during treatment with 500 µM Db-cAMP, pretreated with (black circles) or without (white circles) 20 µM PKI. (**B**) Normalized dye uptake rate in control HeLa Panx1 cells and cells pretreated with 20 µM PKI, 10 µM bisindolylmaleimide (BIM I), 10 µM SB203580, 10 µM AKTi or 10 µM KT5823, subjected to mechanical stretch and subsequently treated with 500 µM Db-cAMP. Statistical analysis in B was performed between each experimental group (connecting lines) and comparing each group with baseline (symbols above each bar): *** *p* < 0.001 and n.s., not significant. Each value in B corresponds to the average ± standard error of a total of three to five independent experiments.

Since PKA is activated by an increase in the cytoplasmic concentration of cAMP [36], and cAMP analogs inhibit the activity of Panx1 channels (see above), we wanted to assess whether PKA signaling pathways are involved in these mechanisms. To address this question, we performed DAPI uptake experiments in HeLa Panx1 cells pretreated for 30 min with 20 µM PKI, a specific PKA inhibitor [39]. Pretreatments with PKI did not affect the mechanical stretch-induced activation of Panx1 channels (Figure 3A), and the application of 500 µM Db-cAMP reduced the rate of DAPI uptake only in HeLa Panx1 cells not treated with PKI (Figure 3A).

Interestingly, the preincubation of HeLa Panx1 cells for 30 min with 500 µM Db-cAMP prevented the mechanical-stretch induced activation of Panx1 channels (Supplementary Figure S2). The average rates of DAPI uptake from three independent experiments are plotted in Figure 3B, showing that PKI treatment did not affect the mechanical stretch-induced activation of Panx1 channels, but rather blocked the inhibitory effect of the cAMP analog.

To evaluate whether the inhibitory effect of cAMP analogs depends on other protein kinases, changes in fluorescence intensity were assessed in the presence of Db-cAMP in HeLa Panx1 cells pretreated for 30 min with different inhibitors—PKC (10 µM bisindolylmaleimide, BIM I), p38 MAPK (10 µM SB203580), Akt (10 µM AKTi), or PKG (10 µM KT5823). None of these four compounds prevented the inhibitory effect of Db-cAMP on mechanical stretch-induced activity of Panx1 channels (Figure 3B). In fact, the results were similar to those of the control conditions (control: with mechanical stretch ~3.8- and with Db-cAMP ~1.1-fold changes relative to baseline; BIM I: with mechanical stretch ~3.6- and with Db-cAMP ~1.4-fold changes relative to baseline; SB203580: with mechanical stretch ~3.5- and with Db-cAMP ~1.4-fold changes relative to baseline; AKTi: with mechanical stretch ~4.3- and with Db-cAMP ~1.1-fold changes relative to baseline; PKG: with mechanical stretch ~3.6- and with Db-cAMP ~1.5-fold changes relative to baseline). These results suggest that PKA may have a direct effect on the activity of Panx1 channels without any crosstalk with any of the other four other protein kinases studied.

### 2.3. Channels Formed by Panx1 Mutated in T302 or S328 by Alanine Are Not Activated by Mechanical Stretch

**Figure 4:**
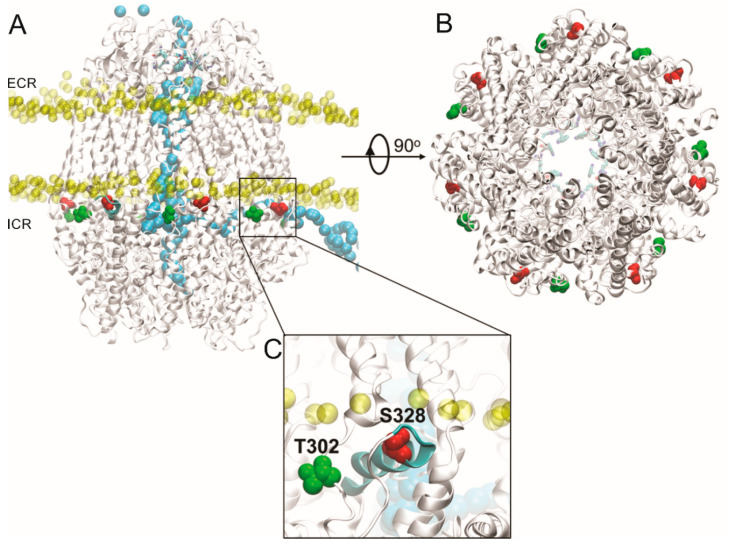
Structural features of putative PKA phosphorylation sites in the Panx1 channel. (**A**) Lateral view of putative PKA phosphorylation sites in Panx1, including the T302 and S328 residues (green and red, respectively), located in the Panx1 C-terminal end. The phosphate atoms of the membrane are shown in yellow. In light blue is shown the trajectory of the Cl^−^ ion crossing the Panx1 channel from the intracellular region, crossing from the lateral tunnel, which is in the inter-subunit interphase, until the main cavity of the pore, then passing until the extracellular region, crossing the selectivity filter (residues W74 and R75 in van der Waals representation). Configuration extracted from molecular dynamics (MD) simulation trajectory. The MD simulation was done using the AMBER force field under an external electric field of ±100 mV. ICR: intracellular region; ECR: extracellular region. (**B**) Intracellular view of the Panx1 channel pore, where the residues of the selectivity filter (show in sticks representation) are observed in the background in blue. The residues S328 and T302 of each segment in the intracellular region of Panx1 are shown in red and green, respectively. (**C**) Close-up view of S328 residue (in red) reveals that it is part of an alpha helix amphiphilic molecule (cyan), which is located in the lipid–water interphase, while the T302 residue (in green) is near to S328, which is inserted into a loop segment.

Based on our findings, we propose that PKA directly phosphorylates Panx1, and that this modification inhibits the mechanical stretch-induced activation of Panx1 channels. To evaluate this hypothesis, we performed a bioinformatic search of putative PKA phosphorylation sites in Panx1 (see Materials and Methods). Four putative PKA phosphorylation sites were found in Panx1 predictively located in intracellular domains: threonine 21 (T21, located at the N-terminal end), serine 205 (S205, located in the intracellular loop), and threonine 302 and serine 328 (T302 and S328, both located in the C-terminal end) (Figure 4).

Additionally, we evaluated the behavior of these residues through MD simulations, finding that the S328 residue is located on the alpha helix segment in the water–lipid interphase, together with the T302 residue. Subsequently, we designed PCR primers to perform alanine substitutions on these residues to prevent phosphorylation (see Table 1 and Table 2, Material and Methods).

**Figure 5:**
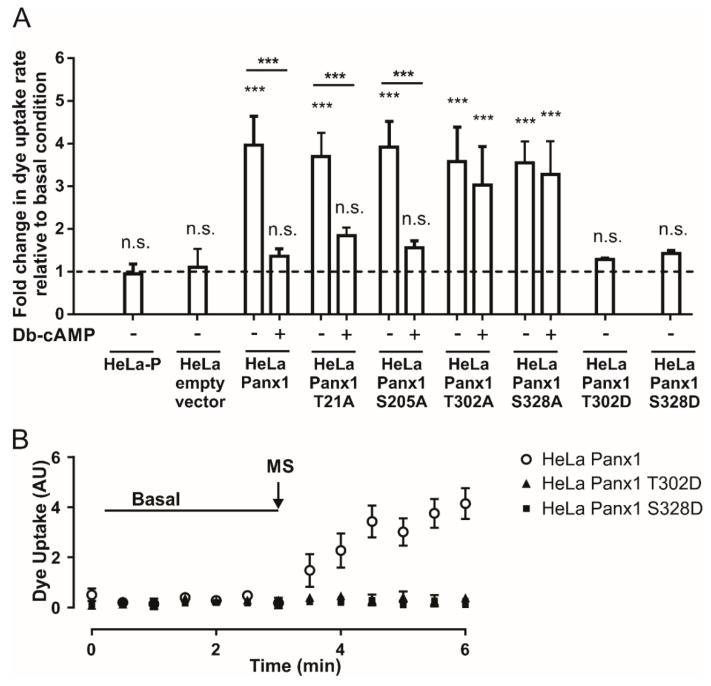
Phosphorylation in threonine 302 or serine 328 is necessary for the inhibition of Panx1 channels induced by Db-cAMP. (**A**) Normalized dye uptake rate in HeLa-P, HeLa-P transfected with pRK5 containing EGFP cDNA (EGFP empty vector), HeLa-Panx1-EGFP, or Hela transfected with Panx1 mutants (HeLa T21A, HeLa S205A, HeLa T302A, HeLa S328A, HeLa T302D and S328D) fused to EGFP subjected to mechanical stretch and subsequently treated with Db-cAMP. (**B**) Temporary course of DAPI uptake in HeLa-Panx1 cells transfected with Panx1-EGFP, Panx1 T302D-EGFP (Hela PanxT302D), or Panx1 S328D-EGFP (HeLa Panx328D) at basal condition and after mechanical stretch (MS, arrow). The dye uptake assay was performed 24 h post-transfection. Statistical analysis in (**A**) was performed between each experimental group (connecting lines), comparing each group with the baseline (symbols shown in each bar). Note: *** *p* < 0.001; n.s., not significant. Each value in (**A**) corresponds to the average ± standard error of a total of three to seven independent experiments.

HeLa-P cells were transfected with the mutant constructs, and 24 h later presented similar cellular distribution to HeLa-Panx1. Western blot analysis showed that the main bands of all mutants co-migrate with Panx1 (Supplementary Figures S3 and S4, respectively). At this time, the activity of the Panx1 channels was evaluated by DAPI uptake assays. As a negative control, we used HeLa-P cells or HeLa-P cells transfected with pRK5 vector containing only the EGFP insert (EGFP empty vector). HeLa-P cells or those transfected with the empty vector were insensitive to mechanical stretch (Figure 5A). In the case of cells transfected with the construct containing Panx1-EGFP, the changes in the rate of DAPI uptake were comparable to those obtained in previous experiments, since cells subjected to mechanical stretch showed a ~4-fold increase in comparison to baseline. In addition, cells subjected to mechanical stretch and then treated with Db-cAMP showed only a ~1.3-fold increase in DAPI uptake rate relative to baseline (Figure 5A).

T21A-EGFP and S205A-EGFP mutants showed similar results, since after mechanical stretch we observed ~3.7- and ~3.9-fold increases, respectively, in the rates of DAPI uptake relative to basal condition. Application of Db-cAMP reduced the DAPI uptake rate to ~1.8- and ~1.5-fold relative to basal condition in T21A-EGFP and S205A-EGFP mutants, respectively (Figure 5A). Regarding cells transfected with T302A-EGFP or S328A-EGFP mutants, the mechanical-stretch induced activity of Panx1 channels was comparable to that of cells transfected with Panx1-EGFP, reaching ~3.6- and ~3.5-fold changes, respectively, in the rate of DAPI uptake relative to basal condition. However, application of Db-cAMP to the T302A-EGFP or S328A-EGFP mutants induced ~3- and ~3.3-fold changes, respectively, in the DAPI uptake rate relative to basal condition (Figure 5A). These results suggest that phosphorylation in either T302 or S328 is necessary for the observed Db-cAMP-dependent inhibition of Panx1 channel activity.

To verify the latter, we generated phosphomimetic Panx1 mutants T302D-EGFP and S328D-EGFP in order to simulate constitutive phosphorylation of these amino acid residues. This was accomplished by using the same strategy described above, utilizing the primers listed in Table 1. In column 2 of Table 2, which corresponds to the DNA sequences of wild-type and mutated segments, the codons that originally encode for threonine or serine and codon (GAC), which encodes for aspartate, are noted in red boxes. In the third column of the same table, the changes in the amino acid residue are presented in red letters, either from threonine or serine to aspartate. HeLa-P cells were transfected with the mutant constructs and 24 h later the activity levels of Panx1 channels were tested. Figure 5B shows representative time lapse experiments of DAPI uptake in HeLa cells transfected with Panx1-EGFP and Panx1 T302D or S328D fused to EGFP. Upon mechanical stretch stimulation, only Panx1-EGFP showed an increase in the DAPI uptake rate. Figure 5A shows the average rate of DAPI uptake relative to basal condition in HeLa cells transfected with Panx1 T302D-EGFP or Panx1 S328D-EGFP after mechanical stretch stimulation. T302D-EGFP and S328D-EGFP mutants showed ~1.3- and ~1.4-fold increases in DAPI uptake rate after mechanical stretch stimulation, respectively, versus a ~4-fold increase relative to basal condition observed in cells transfected with Panx1-EGFP. The rates of DAPI uptake of these mutants did not show any significant differences with respect to baseline uptake. These results are consistent with the proposal that phosphorylation in T302 or S328 favors a low-activity state of Panx1 channels, and therefore strongly suggests that PKA-dependent phosphorylation of at least one of these residues would be sufficient to prevent a mechanical-stretch induced increase in Panx1 channel activity.

## 3. Discussion

In this work, we showed that adenosine inhibits the permeability to DAPI through Panx1 channels expressed in HeLa cells. Accordingly, this response was also dependent on changes in the intracellular concentration of cAMP and PKA activity. The possible role of other protein kinases in reducing the permeability of Panx1 channels to DAPI was ruled out using a pharmacologic approach. Because of the above, we inquired whether PKA directly phosphorylates Panx1 channels to inhibit their activity. The putative PKA phosphorylation sites T21, S205, T302, and S328 in Panx1 were identified in cytoplasmic domains and replaced by alanine, a non-phosphorylatable and neutral residue. In dye uptake experiments, only T302A and S328A mutants were resistant to the inhibitory effect of Db-cAMP, consistent with the interpretation that phosphorylation in T302 or S328 is necessary for the inhibition of the Panx1 channel activity induced by the cAMP analogs. In addition, phosphomimetic mutants T302D and S328D produce Panx1 channels that are insensitive to mechanical stretch stimulation (Figure 5), maintaining the channels with low constitutive activity. Although under basal conditions we did not observe a significant decrease in the activity of Panx1 channels in HeLa Panx1 cells pretreated with Db-cAMP compared to untreated cells (Supplementary Figure S2), our results support the hypothesis that Db-cAMP promotes phosphorylation in T302 or S328, maintaining the channels with low activity, since Hela Panx1 cells exhibit minimal dye uptake under basal conditions that is not significantly different from that of Hela-P cells (Supplementary Figure S1). Therefore, it is expected that Db-cAMP treatment did not further reduce the activity of the Panx1 channels under basal conditions.

**Figure 6:**
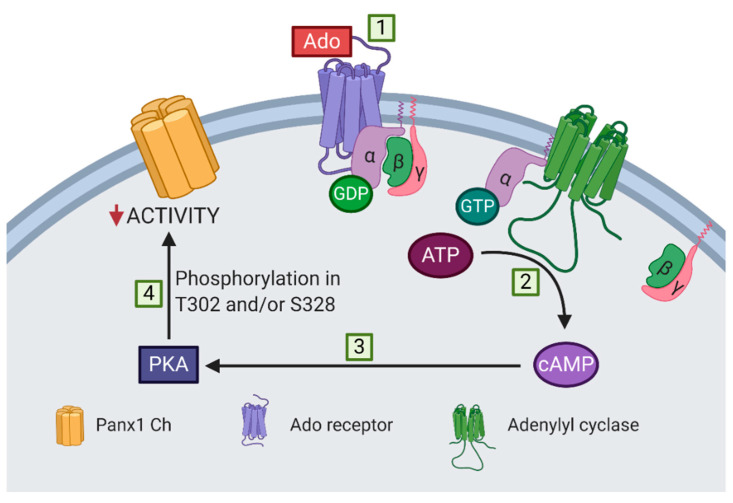
Proposed model of adenosine-dependent inhibition of mechanically stretched Panx1 channels. (1) Adenosine activates receptors associated with the αs subunit, i.e., A_2A_ and A_2B_ receptors, (2) which induce an increase in cytoplasmic cAMP, probably via the activation of adenylyl cyclase. (3) PKA is activated upon an increase in cytoplasmic cAMP, (4) mediating the inhibition of Panx1 channels (Panx1 Ch) through the phosphorylation of T302 or S328 residues.

Figure 6 shows a proposed model of adenosine-induced inhibition of Panx1 channel activity. The activation of adenosine receptors associated with the αs subunit, i.e., A_2A_ and A_2B_ receptors, increases the cytoplasmic cAMP concentration, probably via the activation of adenylyl cyclase. PKA is activated after the increase of cytoplasmic cAMP, mediating the inhibition of Panx1 channel activity through the phosphorylation of T302 or S328. However, the stoichiometry of the phosphorylation of the Panx1 channel remains to be determined; closure of all 7 lateral tunnels might require that each subunit should be phosphorylated in T302 or S328 to obtain the maximal reduction in activity of a Panx1 channel.

In order to evaluate the structural microenvironment of the residues suitable for phosphorylation, we generated a molecular model of the Panx1 channel. Our model revealed that the S328 and T302 residues are located close to the lipid–water interphase, but with greater accessibility to the aqueous solvent. At the same time, these residues are near the lateral tunnels of the intracellular domain of the protein. Figure 4 shows the lateral view of putative PKA phosphorylation sites in Panx1, including the T302 and S328 residues. As a reference, the phosphorus atoms close to the head of the membrane lipids are shown. A selected trajectory of a chloride ion (Cl^−^) is represented in Figure 4, with the purpose of showing the translocation process. Interestingly, the Cl^−^ ion selected in this picture shows the translocation pathway through the Panx1 channel from the intracellular region, which passes through the lateral tunnel, which is in the inter-subunit interphase, until the main cavity, then it moves until the selectivity filter in the extracellular region. That crevice forms a tunnel that connects the main pore with the cytoplasmic region. Another interesting observation is that the S328 residue is part of an amphiphilic alpha helix located in the lipid–water interphase. At the same time, the T302 residue is near to S328 as part of a loop. Therefore, the phosphorylation of any of these residues could promote a structural perturbation of the alpha helix of the S328, losing the secondary structure of this section or perturbing the so-called lateral tunnel [4] of the intracellular region, decreasing the translocation probability through Panx1 channels.

Our results do not distinguish whether adenosine and cAMP reduce the open probability (time that the channel remains open) or single-channel permeability of Panx1 channels, or if they modify the traffic turnover and permanence of Panx1 in the plasma membrane. These questions could be answered by using excised patches to study the open probability and unitary conductance of Panx1 channels (keeping in mind that permeability changes might occur without significant changes in single-channel conductance), and using confocal microscopy experiments with adenosine and cAMP analogs treatments to study Panx1 trafficking. Although membrane stretching has been shown to activate Panx1 channels [6], possibly through a mechanism that involves an interaction of the C-terminus with F-actin [40], this mechanism has also been challenged, since Panx1 channels were not activated by swelling [30]. Despite this controversy, the mechanism by which PKA phosphorylation prevents the mechanical stretch from increasing the activity of Panx1 channels remains to be elucidated.

The involvement of PKA in regulating the permeability to DAPI of Panx1 channels was supported by the following findings: (1) the DAPI uptake rate was reduced by membrane-permeant activators of PKA (cAMP analogs); (2) the DAPI uptake rate was reduced in seconds after exposure to cAMP analogs, supporting the notion that this could be due to a post-translational modification, such as phosphorylation rather than transcriptional regulation; (3) pretreatments with a PKA inhibitor blocked the effect of cAMP analogs on the DAPI uptake rate; (4) the effects of cAMP analogs were not prevented by inhibition of PKC, p38 MAPK, Akt, or PKG kinases; (5) a phosphomimetic mutation of Panx1 in either of the two putative PKA phosphorylation sites maintained Panx1 channels with low activity. These pieces of evidence are very interesting, since a mechanism of Panx1 channel closure in HEK 293 cells transfected with Panx1 cDNA has been described, whereby the treatment with a nitric oxide donor activates PKG, which phosphorylates Panx1 in serine 206 and induces the closure of these channels [35]. However, our results show that cAMP acts through a different mechanism than nitric oxide, since KT5823, a PKG inhibitor, did not prevent cAMP analog- induced inhibition of Panx1 channels. These results establish a new mechanism of PKA-mediated regulation of Panx1 channel activity, whereby the inhibition of the channels occurs by phosphorylation in T302 or S328. To give perspective, it could be studied how PKA-mediated phosphorylation of Panx1 is reversed upon dephosphorylation. Possible phosphatases that could be involved are those of the phosphoprotein phosphatase family, comprising PP1, PP2A, PP2B, PP4, PP5, PP6, and PP7. Among these, PP1 and PP2A can dephosphorylate connexin 43 [41]; therefore, it could be hypothesized that they also could target Panx1 and reverse PKA inhibition of Panx1 channel activity.

G_αq/11_-linked G- protein coupled receptor (GPCR) signaling has been related to the activation of Panx1 channels, since activation of α1D receptors by phenylephrine (in the vasoconstriction response of resistance arteries) and of the PAR-1 receptor by thrombin induces the secretion of ATP through Panx1 channels [42,43,44]. There is similar evidence of Panx1 channel activation through the metabotropic P2Y receptor. In this case, Panx1 channels are activated by an increase in intracellular Ca^2+^ through phospholipase C signaling [44]. Despite the above, to our knowledge, there was no evidence of Panx1 channel modulation by G_αs_-linked GPCR signaling. Therefore, the effect of adenosine acting probably through its G_αs_-linked receptors on Panx1 channels was investigated, and inhibition thereof was observed. However, this regulatory mechanism could eventually occur after the activation of different GPCRs associated with the αs subunit. In this context, it would be interesting to study whether the stimulation of different G_αs_-linked GPCR receptors also induces the inhibition of Panx1 channels, for example some neurotransmitter receptors such as the D1 dopamine receptors, β adrenergic receptors, and 5-HT_4/6/7_ serotonin receptors [45,46,47], hormone receptors such as glucagon [48], and molecule receptors such as histamine H2 [49]. Conversely, stimulation of G_αi/o_-linked GPCR receptors such as α_2_ adrenergic receptors, D2 dopamine receptors, and 5-HT_1/5_ serotonin receptors [45,46,47] could lead to an increase in Panx1 channel activity, since they inhibit adenylyl cyclase, cAMP production, and PKA activation.

Our results could have physiological importance for several cell types, for example in CD4^+^ T lymphocyte activation, a condition in which the opening of Panx1 channels occurs, allowing ATP release and autocrine activation of the cells [23], condition that would be favored if dephosphorylation of the PKA sites found in Panx1 occurs. Additionally, it has been established that adenosine prevents the activation of CD4^+^ T cells in an effect mediated by the A_2A_ receptor [50]. Hence, the inhibition of CD4^+^ T lymphocytes by adenosine generated extracellularly from excess ATP release could be at least partially explained by the inhibition of Panx1 channels, as described here, preventing cell activation. Other examples are the Panx1 channels’ participation in purinergic signaling in different cell types (e.g., neurons, astrocytes, palate cells, endothelial cells, erythrocytes, and immune system cells) [6,10,11,51]; the inhibition of these channels by PKA may disturb purinergic signaling and Ca^2+^ wave propagation in different cells types, leading to a protective effect against seizures and epilepsy, Alzheimer’s disease, stroke, major depression, and schizophrenia, which are characterized by neuroinflammatory processes in the central nervous system induced by extracellular ATP and activation of P2 receptors [12,52]. Additionally, inhibition of Panx1 channels could lead to disrupted taste signaling and vasodilatation in response to hypoxia [51,53].

Although inhibition of Panx1 channel activity by a higher extracellular ATP concentration than that required for P2X7R activation could be sufficient for channel regulation [54], our results may explain a more complex regulation system driven by extracellular ATP. Active Panx1 channels permit the release of ATP to the extracellular milieu, which activates P2 receptors and subsequently activates Panx1 channels, establishing a feed-forward mechanism of ATP release. To regulate this response, ectonucleotidase CD39 could degrade ATP to AMP and ectonucleotidase CD73 could degrade AMP to adenosine [55]. The latter could activate P1 receptors A_2A_ and A_2B_, activating the signaling pathway exposed here to inhibit Panx1 channel activity.

## 4. Materials and Methods

### 4.1. Reagents

Carbenoxolone (CBX), adenosine, 8-CPT-cAMP, Db-cAMP, PKI, SB203580, and AKTi were from Sigma-Aldrich (Saint Louis, MO, USA). 4′,6-Diamidino-2-phenylindole (DAPI) was from Invitrogen, Dulbecco’s modified Eagle’s medium (DMEM) and RPMI 1640 culture medium were from Gibco, and TurboFect reagent was from Thermo Scientific, the three brands belonging to Thermo Fisher Scientific (Waltham, MA, USA) and bisindolylmaleimide (BIM I) was from Cayman Chemical (Ann Arbor, MI, USA).

### 4.2. Cell Lines

Parental HeLa cells (HeLa-P) were obtained from the American Type Culture Collection (ATCC, Rockville, MD) and cultured as described in [37]. HeLa cells transfected with the Panx1 cDNA fused to EGFP (HeLa Panx1) were donated by Dr. Felixas Bukaukas (Department of Neuroscience, Albert Einstein College of Medicine, Bronx, NY, USA). Both cell types were grown in DMEM medium. Jurkat cells were kindly provided by Dr. Mauricio Henríquez of the University of Chile and were grown in RPMI 1640 medium. All cell types were grown in the corresponding medium supplemented with 10% fetal bovine serum and 50 U/mL penicillin–streptomycin, maintained at pH 7.4 at 37 °C in an incubator with a 5% CO_2_/95% air atmosphere. To maintain stable transfection of HeLa Panx1 cells, they were cultured with 1 mg/mL G418 (Invitrogen, Waltham, MA, USA).

### 4.3. Plasmids

The previously described plasmid pRK5 rPanx1 EGFP, encoding *Rattus norvegicus Panx1* tagged with enhanced green fluorescent protein (*EGFP*) reporter gene (Panx1-EGFP) [56] was kindly provided by Dr. Roberto Bruzzone, Institute Pasteur and the University of Hong Kong. The control EGFP empty plasmid pRK5 was generated by digestion of the plasmid pRK5 rPanx1 EGFP with EcoRI restriction enzyme (New England Biolabs, Ipswich, MA, USA), for which there is a cutting site at the multicloning site upstream of the rPanx1 insert and towards the end of the insert itself. Once the plasmid was ligated, the open reading frame for EGFP did not change, and the cells transfected with plasmid pRK5 were detected by their green fluorescence. Plasmids containing the different Panx1 mutations fused to EGFP were generated using the Phusion Site-Directed Mutagenesis Kit (Thermo Scientific, Waltham, MA, USA), as explained below.

### 4.4. Mechanical-Stretch Induced Activation of Panx1 Channels

Although Panx1 channels are not intrinsically mechanosensitive [30], their indirect response can be used to promote their activity, as described previously [57]. Thus, we calibrated a method to induce the activation of Panx1 channels by mechanical stretch. For this, dye uptake experiments were performed in HeLa Panx1 cells grown on glass coverslips, in which fields were randomly selected and the basal fluorescence intensity of DAPI in the nuclei was measured. Subsequently, different volumes of recording solution were dropped onto the cells (1–8 mL) in drip form from 10 cm high, observing a linear relationship between the volume of the mechanical stretch and the increase in the uptake of DAPI, giving a maximum effect from the stimulus of 6 mL. This was inhibited with 10 µM carbenoxolone (CBX), a blocker of Panx1 channels (Supplementary Figure S1).

### 4.5. Evaluation of Pannexin 1 Channel Activity

HeLa Panx1 cells were seeded on 25 mm diameter glass coverslips with 25% confluence, then 24 h later the permeability tracer uptake test was performed to evaluate the activity of Panx1 channels. For this, the coverslips were transferred to a recording chamber, then filled with Krebs solution (in mM: 118 NaCl; 4.7 KCl; 1.2 KH_2_PO_4_; 1.2 MgSO_4_; 4.2 NaHCO_3_; 2 CaCl_2_; 10 glucose and 10 HEPES; pH 7.4) and 5 μM DAPI. The regions of interest corresponding to the cell nuclei were selected and the fluorescence intensity values over time were measured. The images were captured every 30 s using an inverted fluorescence microscope (Eclipse Ti, Nikon, Melville, NY, USA) and NIS Elements control software (Nikon, Melville, NY, USA). Subsequently, the fluorescence intensity was analyzed using the same software. Records of 5 min were obtained for each condition. The dye uptake rates for each period of time or condition were compared, corresponding to the intensity of fluorescence in arbitrary units (AU) divided by the time in minutes (AU/min). The uptake rate of each condition was normalized to the basal uptake rate. The fold changes relative to basal condition were shown (normalized dye uptake rate).

### 4.6. Transfections

TurboFect was used for all transfections, according to the manufacturer’s instructions. In 400 μL of DMEM, 4 μg of each plasmid was resuspended and 6 μL of TurboFect reagent was added. The mixture was incubated for 20 min at room temperature and subsequently added by dripping onto HeLa P cells that had been seeded in glass coverslips of 25 mm diameter at 50% confluence.

### 4.7. Bioinformatics Search of Putative Phosphorylation Sites in Panx1 by PKA

To determine the putative phosphorylation sites of Panx1 in the different states of the channel and the kinases involved in each process, a bioinformatic search was performed using NetPhosK 3.1, GPS 3.0, Kinase Phos, and PhosphoSitePlus software, v6.5.9.3, which are available on the internet. To determine the locations of the different amino acid residues found, we used the Protter Visualize Proteoforms software [58]. Then, we filtered them to keep only the amino acid residues that would be located in the intracellular region and that are putatively phosphorylatable by PKA. The search yielded the following results: N- terminal T21, S205 located in the intracellular loop, and C- terminal amino acid residues T302 and S328.

A full model of the Panx1 channel (sequence code Q96RD7 (UniProt)) was built using the Modeller program to complete the lateral chains and fragments lost in the Cryo-EM wild-type human Panx1 channel (PDB: 6WBF). A molecular dynamics (MD) simulation of wild-type Panx1 protein was performed to evaluate the translocation events of the K^+^ and Cl^−^ when the residues were phosphorylated. The human Panx1 channel structure was refined and inserted into the phosphate atoms of a membrane measuring 180 × 180 Å. The water (TIP3) box was added, neutralized, and a concentration of 0.15 mol/L of KCl was added. The system was submitted to energy minimization until convergence, then subsequently an equilibration protocol was run for 200 ns using AMBER 18 software [59], using a constant number of particles, constant pressure, and constant temperature (310 K). MD simulation was carried out to calculate the rate of K^+^ and Cl^−^ ion permeation through the Panx1 channel. In order to characterize the ion pathway translation, the protein was submitted to an external electric field of ±100 mV during 500 ns of MD simulation using a time step of 2 fs to drive ions through the channel. All figures were constructed using VMD software [60].

### 4.8. Point Mutations

Mutations of the amino acid residues described above (T21, S205, T302, and S328) were made from an alanine or aspartate residue (Table 1). For this, pairs of specific PCR primers were designed, containing the mutagenic sequence in the forward primer. The mutations and primers are shown in Table 2**.**

Table 1 shows segments of the wild-type and mutant DNA (second column) and protein sequences (third column). Codons that originally encode threonine (ACC) or serine (TCC) and mutated codons that encodes alanine (GCC) or aspartate (GAC) are highlighted in red boxes in the second column. Targeted threonine (T) or serine (S) residues substituted by alanine (A) or aspartate (D) are highlighted in red in the third column of the same table.

We carried out the mutations utilizing the Phusion Site-Directed Mutagenesis Kit, using as template DNA the plasmid pRK5, containing an insert of rat Panx1 fused to EGFP (pRK5-rPanx1 EGFP).

The codons that generate each mutation are highlighted with bold letters. It is worth mentioning that these primers were designed manually and were synthesized by Integrated DNA Technologies (IDT, Skokie, IL, USA), containing 5′ phosphorylated ends. Mutations were made with the Phusion Site-Directed Mutagenesis Kit (Thermo Scientific, Waltham, MA, USA) according to the manufacturer’s instructions. In summary, a PCR reaction was carried out, using as a template the plasmid pRK5 containing the rat Panx1 insert fused to EGFP (pRK5-Panx1 EGFP), the plasmids corresponding to the mutation to be performed, and the reagents contained in the kit. This reaction allowed us to obtain a product corresponding to the complete linearized plasmid with the mutated Panx1 fused to EGFP.

Subsequently, a ligation reaction was performed using the Rapid DNA Ligation Kit (Thermo Scientific, Waltham, MA, USA), and DH5α chemically competent *E. coli* cells were transformed with the different mutant plasmids, leaving them in LB agar medium (Mo Bio Laboratories, Carlsbad, CA, USA) with 100 μg/mL ampicillin for 24 h in an incubator at 37 °C. Next, 5 colonies were grown for each transformation in LB broth (Mo Bio Laboratories, Carlsbad, CA, USA) with 100 μg/mL ampicillin for 24 h. Then, plasmid DNA was purified from each bacterial culture using the E.Z.N.A. Plasmid Mini Kit II (Omega Bio-tek, Norcross, GA, USA). To verify that the plasmids obtained contained the desired mutations, they were sequenced in the Sequencing Unit of the Pontificia Universidad Católica de Chile with an ABI PRISM 3500 xl sequencer (Applied Biosystems, Foster City, CA, USA). The plasmids that contained the mutations and preserved the open reading frames of Panx1 and EGFP were selected and used to transiently transfect HeLa-P cells.

### 4.9. RT-PCR

To detect A_2A_ and A_2B_ adenosine receptors of mRNA in HeLa Panx1 cells, total RNA was extracted from ~5 million Jurkat cells (positive control) and ~1 million HeLa Panx1 cells using TRIzol reagent (Fisher Scientific, Waltham, MA 02451, USA). The extract was treated with DNase RQ1 RNase-Free (Promega, Madison, WI 53711 USA) to degrade contaminating DNA, and a cDNA was synthesized using the SuperScript First-Strand Synthesis System kit (Invitrogen, Waltham, Massachusetts, USA), according to the manufacturer’s instructions. PCR for GAPDH was performed as a constitutive expression control, using the primer forward 5′ ACCACAGTCCATGCCATCAC 3′ and primer reverse 5′ TCCACCACCCTGTTGCTGTA 3′, obtaining an amplicon of 452 bp; for the A_2A_ adenosine receptor, using the forward primer 5′ TGCAGAACGTCACCAACTAC 3′ and the reverse primer 5′ GCCAGGAAGATCCGCAAATA 3′, obtaining a 499 bp amplicon; for the A_2B_ adenosine receptor, using the forward primer 5′ TGTGTCCCGCTCAGGTATAA 3′ and the reverse primer 5′ TCGGTTCCGGTAAGCATAGA 3′, obtaining an amplicon of 564 bp. In all cases, the reaction without template DNA was used as a negative control. The primers were synthesized by Integrated DNA Technologies (IDT, Skokie, IL, USA).

### 4.10. Statistical Analysis

Dye uptake rates are shown as the average dye uptake divided by time ± standard error, normalized to the baseline dye uptake rate. Significant differences between groups were determined using two-way ANOVA with a Tukey post hoc test to perform multiple comparisons. The differences were considered significant at *p* < 0.05 (* *p* < 0.05, ** *p* < 0.005, *** *p* < 0.001). The number of repetitions for each experiment is mentioned in the description of each figure.

## Figures and Tables

**Table 1:** Sequences of alanine and aspartate mutations of putative phosphorylation sites of Panx1 by PKA.

Mutation	DNA Segment	Protein Segment
rPanx1 rPanx1 T21A	GAGCCC**ACC**GAGCCC GAGCCC**GCC**GAGCCC	FLLKEPTEPKFKG FLLKEPAEPKFKG
rPanx1 rPanx1 S205A	AAGAA**TCC**AGTCAC AAGAA**GCC**AGTCAC	LKTKKNSSHLIMK LKTKKNASHLIMK
rPanx1 rPanx1 T302A	CAGAAG**ACG**GACGTC CAGAAG**GCC**GACGTC	VPFRQKTDVLKVY VPFRQKADVLKVY
rPanx1 rPanx1 T302D	CAGAAG**ACG**GACGTC CAGAAG**GAC**GACGTC	VPFRQKTDVLKVY VPFRQKDDVLKVY
rPanx1 rPanx1 S328A	GACTTG**AGC**CTCTAC GACTTG**GCC**CTCTAC	EGYNDLSLYNLFL EGYNDLALYNLFL
rPanx1 rPanx1 S328D	GACTTG**AGC**CTCTAC GACTTG**GAC**CTCTAC	EGYNDLSLYNLFL EGYNDLDLYNLFL

Bold letters indicate the differences in nucleotide sequence corresponding to wild type rPanx1 and rPanx1 mutants. In red are indicated the amino acid residues that are modified in each mutant compared to the one shown right on top present in wild type rPanx1.

**Table 2:** PCR primers designed to perform site-directed mutations of amino acid residues of rPanx1.

Mutation	Primers
**T21A**	**Forward** GGAGCCC**GCC**GAGCCCA **Reverse** TTCAGCAAGAAGTCCGAGAACAC
**S205A**	**Forward** CGAAGAAGAAC**GCC**AGTCACCTAAT **Reverse** TCTTCAAGTACTGCTCCACGATC
**T302A**	**Forward** GGCAGAAG**GCC**GACGTCCT **Reverse** GGAACGGGACGAAGAGCGT
**T302D**	**Forward** GGCAGAAG**GAC**GACGTCCT **Reverse** GGAACGGGACGAAGAGCGT
**S328A**	**Forward** CAACGACTTG**GCC**CTCTACAACC **Reverse** TAGCCTTCAGACTTGAAATGTAGAACATC
**S328D**	**Forward** CAACGACTTG**GAC**CTCTACAACC **Reverse** TAGCCTTCAGACTTGAAATGTAGAACATC

Each set of three bold letters correspond to a codon designed to introduce the desired mutation.

## Supplementary Materials

Supplementary Materials can be found at https://www.mdpi.com/1422-0067/21/23/9180/s1.

## Abbreviations

DAPI	4′,6-Diamidino-2-fenilindol
Panx1	Pannexin 1
cAMP	Cyclic Adenosine monophosphate
PKA	Protein kinase A
PKC	Protein kinase C
PKG	Protein kinase G
ATP	Adenosine triphosphate
