# Supplementary material for: Stretch-Induced Activation of Pannexin 1 Channels Can Be Prevented by PKA-Dependent Phosphorylation
Source: Int J Mol Sci. 2020 Dec 2;21(23):9180. doi: 10.3390/ijms21239180 (PMC7731223; doi:10.3390/ijms21239180)
Supplement: Supplementary file 1 [file ijms-21-09180-s001.pdf]

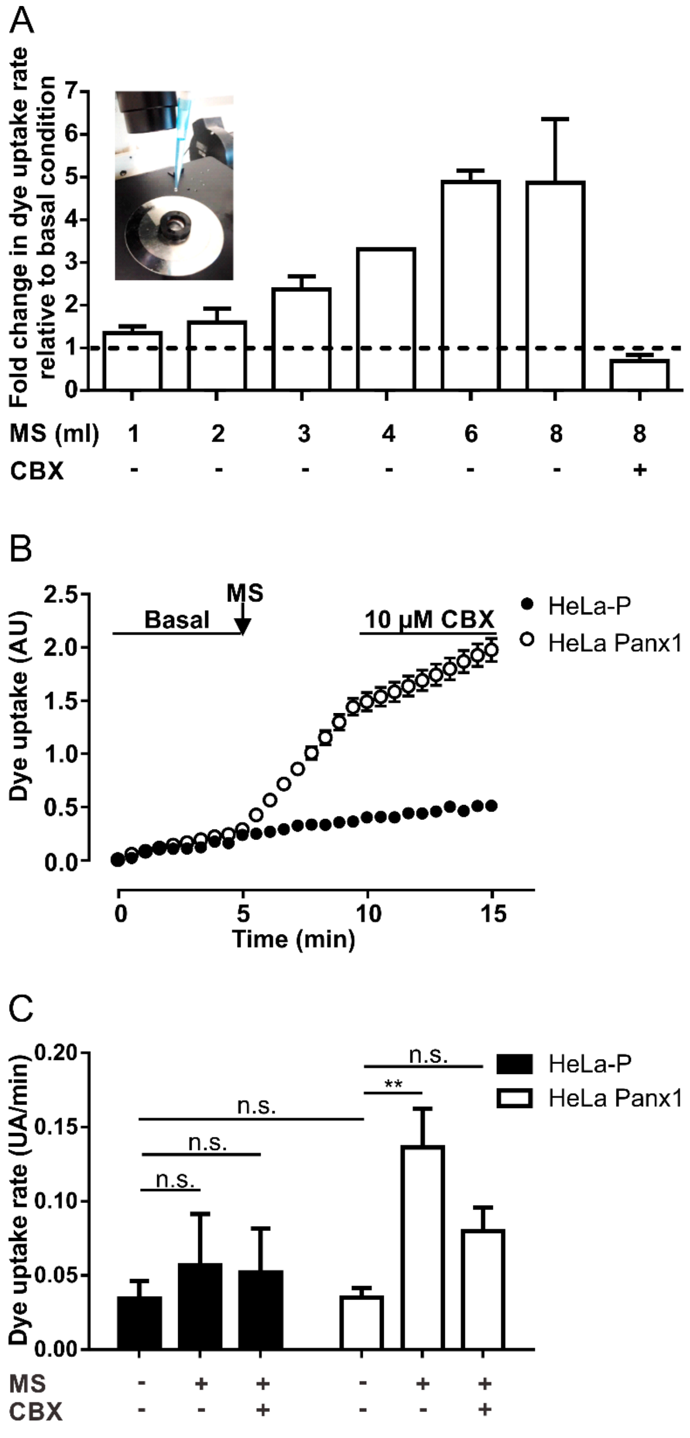

Supplementary Figure 1: Calibration of Panx1 channel activation by mechanical stretch. HeLa Panx1 cells were seeded on 25 mm diameter glass coverslips with 25% confluency and 24 h later they were transferred to a recording chamber with 5 μM DAPI in Krebs solution. **(Insert in A)** Picture of the experimental setup. Basal DAPI uptake was recorded for 5 min and subsequently, increasing volumes of recording solution were taken by a p1000 micropipette and dropped onto the chamber (1 to 8 ml), registering 5 min of each condition. **(A)** Normalized dye uptake rate in HeLa Panx1 cells subjected to mechanical stretch (MS) by dropping increasing volumes of recording solution (Column 1 to 6 in ml: 1, 2, 3, 4, 6 and 8 respectively). Column 7: Upon 8 ml treatment, cells were treated with 10 μM CBX. **(B)** Temporary course of DAPI fluorescence intensity measured in arbitrary units (AU) in HeLa-P (black circles) and HeLa Panx1 (white circles) cells at basal condition, after MS induced by 6 ml of extracellular solution, and after treatment with 10 μM carbenoxolone (CBX). **(C)** Dye uptake

rate in HeLa-P cells (black columns) and HeLa Panx1 (white columns) under the same conditions shown in B. Statistical analysis was performed comparing between groups: * p <0.05, ** p <0.005 and n.s., not significant. Each value corresponds to the average ± standard error of a total of three independent experiments.

Appendix B

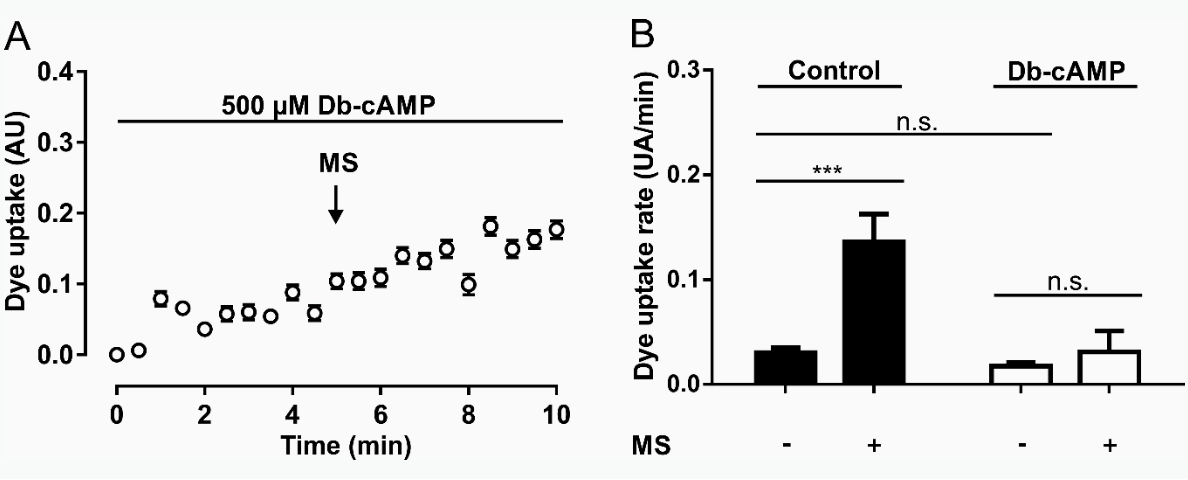

Supplementary Figure 2: Db-cAMP pretreatment prevents mechanical stretch -dependent activity of Panx1 channels. (A) Temporary course of DAPI fluorescence intensity in HeLa Panx1 cells pretreated for 30 min with 500 µM Db-cAMP and subjected to mechanical stretch (MS). Besides Db-cAMP pretreatment, the experiment was carried out in the presence of 500 µM Db-cAMP. **(B)** Dye uptake rate in HeLa Panx1 cells pretreated (white bars) or not pretreated (black bars) for 30 min with 500 µM Db-cAMP and subjected to MS (indicated by arrow). Statistical analysis was performed comparing between groups: * p <0.05, ** p <0.005 and n.s., not significant. Each value corresponds to the average ± standard error of a total of three independent experiments.

Appendix C

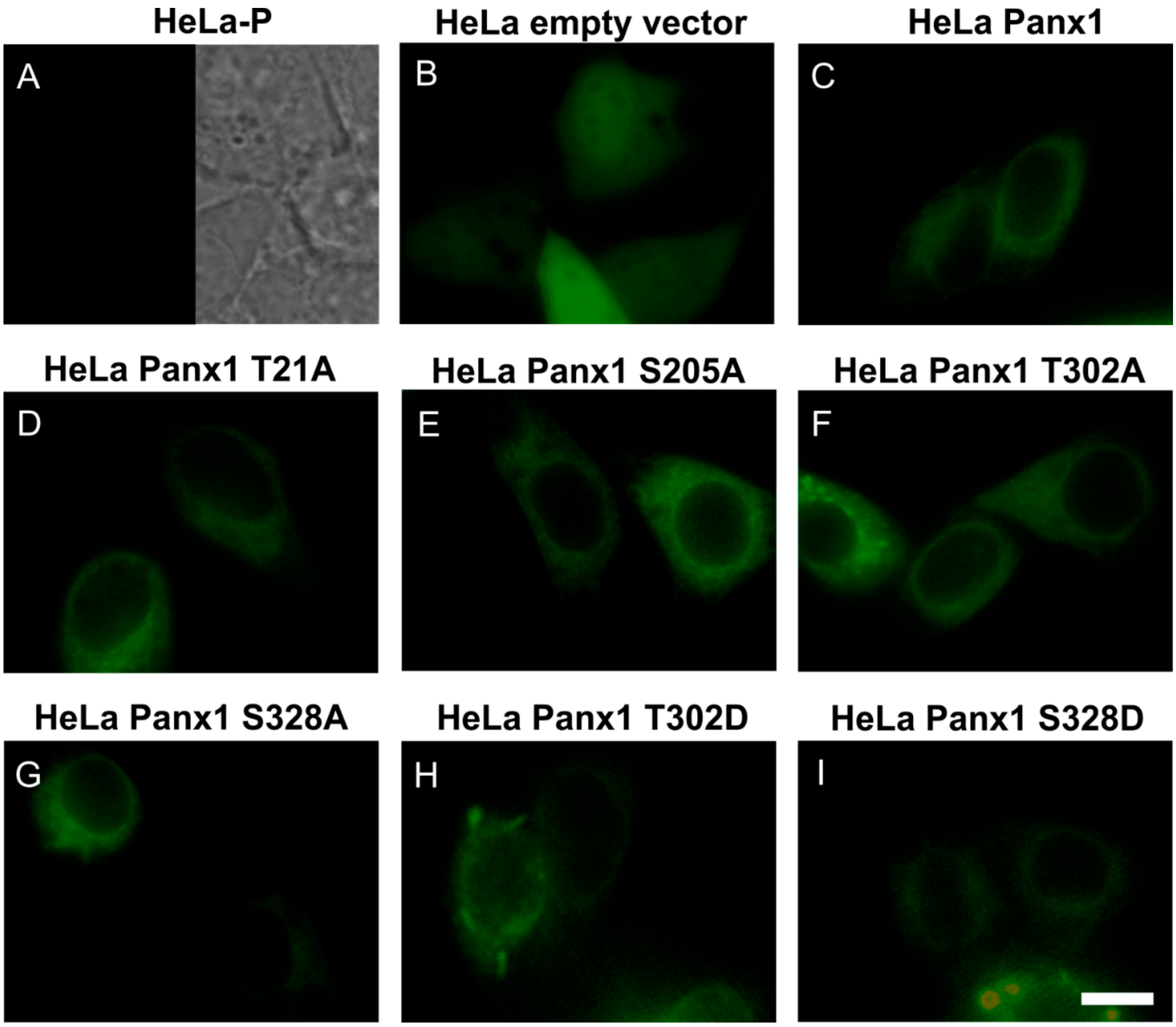

Supplementary Figure 3: Expression and distribution of Panx1 and Panx1 mutants in HeLa cell transfectants. Panx1-EGFP fluorescence in HeLa-P and HeLa cells transfected with empty vector, Panx1 and Panx1 mutants. HeLa –P cells were seeded on glass covers and 24 h later they were transfected with different DNA constructs. Then, 24 h after transfection EGFP fluorescence was observed in order determine the expression of Panx1. Representative fluorescent fields showing Panx1-EGFP fluorescence are shown in panels A-I. HeLa-P cells are shown in panel A showing the EGFP channel on the left and bright view of the same field on the right. Scale bar: 10 μm.

Appendix D

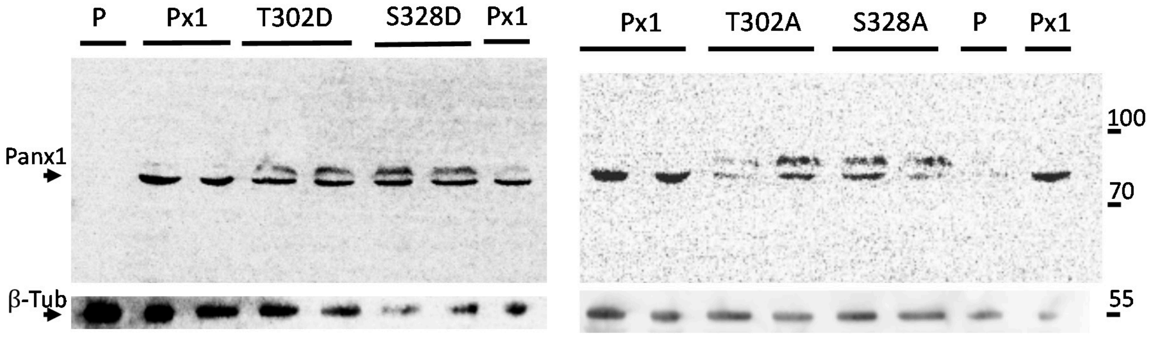

Supplementary Figure 4. Western blot of Panx1 and Panx1 mutants transfected in HeLa cells. Parental HeLa cells (P), HeLa Panx1 (Px1) and HeLa cells transfected with mutants T302D, S328D, T302A, or S328A were homogenized and 65 μg of total protein were resolved in 12.5% SDS polyacrylamide gels and electro transferred to nitrocellulose sheets. Panx1 and mutants were detected with anti-Panx1 polyclonal antibody described previously [24]. Blots were rinsed with PBS solution–1% Tween 20 and incubated for 20 min at room temperature with HRP-conjugated goat anti-rabbit or anti-mouse IgGs (Santa Cruz Biotechnology). After several rinses, immunoreactive proteins were detected using ECL reagents according to the manufacturer's instructions (PerkinElmer) [24]. β-Tubulin was developed and served as loading gel control. Number on the right side of the gel are approximate molecular mass of standard markers.
